# A prospective randomized radiographic and dual-energy X-ray absorptiometric study of migration and bone remodeling after implantation of two modern short-stemmed femoral prostheses

**DOI:** 10.1007/s10195-015-0335-1

**Published:** 2015-02-11

**Authors:** Volker Brinkmann, Florian Radetzki, Karl Stefan Delank, David Wohlrab, Alexander Zeh

**Affiliations:** Department of Orthopaedics and Traumatology, Faculty of Medicine, Martin-Luther-University of Halle-Wittenberg, Ernst-Grube-Strasse 40, 06120 Halle/Saale, Germany

**Keywords:** Metha™, Nanos™, Short-stemmed prosthesis, DEXA, Stress shielding

## Abstract

**Background:**

The aim of this prospective randomized study was to analyze migration and strain transmission of the Metha™ and Nanos™ femoral prostheses.

**Materials and methods:**

Between 1 January 2011 and 2 April 2013, 50 patients were randomized to receive short-stemmed femoral prostheses. Metha™ stems were implanted in 24 patients (12 female, 12 male; mean age 58.7 years; mean body mass index [BMI] 27.4) and Nanos™ stems in 26 patients (10 female, 16 male; mean age 59.7 years; mean BMI 27.1). Longitudinal stem migration, varus−valgus alignment, changes of center of rotation (COR), femoral offset and caput-collum-diaphyseal angle, leg length discrepancy, periprosthetic radiolucent lines incidence, and dual-energy X-ray absorptiometry (DEXA) scans were analysed after an average of 98 and 381 days.

**Results:**

There was no significant change of varus−valgus alignment or clinically relevant migration of the Metha™ or Nanos™ prostheses during postoperative follow-up. After 12.3 months, the DEXA scans showed small but significant differences of bone mineral density in Gruen zones 1 (minus ~8 %) and 6 (plus ~9 %) for the Metha™ and in Gruen zone 1 (minus ~14 %) for the Nanos™ (paired *t* test). Visual analog scale (VAS) and Harris Hip Score (HHS) improved significantly for both implants (Nanos™/Metha™ 12.3 months postoperatively HSS 96.5/96.2; VAS 0.7/0.8, respectively). COR or offset did not change significantly after surgery.

**Conclusions:**

Neither implant showed signs of impaired osseointegration. DEXA demonstrated proximally located load transfer with only moderate proximal stress shielding.

**Level of evidence:**

II.

## Introduction

The use of short-stemmed femoral prostheses in total hip arthroplasty (THA) has increased considerably with the development of several such stems by different manufacturers [1].

There are numerous studies reporting excellent short- and medium-term clinical and radiological results [2–7]. Short-stemmed femoral implants were designed to achieve proximal load transfer in the femoral metaphysis in order to prevent stress shielding and preserve metaphyseal bone. Because of their shape and short design, they are particularly suitable for less invasive approaches [8].

Investigations of load transfer after femoral stem implantation have generally been performed using dual-energy X-ray absorptiometry (DEXA) measurements [2, 3, 5–7, 9, 10], although other study groups favor computed tomography scans [11]. Studies examining strain distribution after implantation of short-stemmed femoral prostheses have yielded conflicting results regarding the achievement of selectively proximal load transfer [5, 6, 12, 13]. Proximal load transfer is considered one major advantage compared to conventional stems, which typically produce clinically relevant stress shielding. It is thus conjectured that short-stemmed prostheses should preserve metaphyseal bone and, in this way, facilitate the eventual exchange to conventional prostheses, e.g., in cases of aseptic loosening [1]. In addition, there is evidence that bone loss around femoral stems might be associated with an increased risk of aseptic loosening [14].

To date, no single published study has concluded that short-stemmed femoral implants show the same excellent long-term survival as conventional cementless stems and/or lead to improved options for revision THA.

The Metha™ (Aesculap AG, Tuttlingen, Germany) non-cemented stem is anchored in the metaphysis within the closed ring of the femoral neck. The conical shape promotes primary stability and proximal force transfer. The good primary stability is further enhanced by the rounded tip of the stem along the dorsolateral cortex. The Plasmapore^®^μ-CaP coating of the entire proximal surface encourages rapid secondary osseointegration. In this study, the Metha™ stem was implanted as a monoblock, which is available with neck angles of 125, 130, and 135° (courtesy of Aesculap AG).

The Nanos™ (Smith & Nephew GmbH, Marl, Germany) prosthesis is designed to affix in the calcar region to ensure optimum load transfer, and to bind along the distal lateral cortex to support and compensate varus loading. The implant is made of a titanium forged alloy (ISO 5832-3), with an osteoconductive proximal coat. The roughness of the titanium plasma surface both increases surface area and ensures superior primary stability. The additional calcium phosphate (BONIT^®^) allows acceleration of the osseointegration process (courtesy of Smith & Nephew GmbH).

This study investigated osseointegration and bone remodeling after implantation of the Metha™ or Nanos™ prostheses, to analyze whether proximal load transfers could be achieved and whether there are differences between the two implants.

## Materials and methods

Between 1 January 2011 and 2 April 2013, 50 patients undergoing THA for severe primary coxarthrosis (Kellgren III or IV) and failed conservative treatment were randomized to receive short-stemmed femoral prostheses. Metha™ stems were implanted in 24 patients (12 female, 12 male) and Nanos™ stems were implanted in 26 patients (10 female, 16 male) (Table 1). Patients >70 years, those receiving cortisone therapy, and those with cancer, rheumatoid arthritis, osteoporosis, and/or other bone or connective tissue diseases were excluded from the study.

**Table 1:** Antropometric data

Parameter	Metha™ group (SD) [min−max]	Nanos™ group (SD) [min−max]
Age (years)	58.7* (7.9) [43/70]	59.7* (6.5) [48/70]#
Height (cm)	172.9* (6.7) [163/189]	172* (8) [156/190]#
Weight (kg)	81.4* (13.1) [56/105]	80.3* (11.5) [60/108]#
BMI (kg/m^2^)	27.4* (4.5) [19/39]	27.1* (2.4) [21/33]#
OP-time (min)	75* (23) [35/111]	69* (23) [30/115]#

* Mean

SD = standard deviation, min–max = minimum–maximum

# Not significant (unpaired *t*-test, *p* > 0.05)

Postoperatively, all patients were mobilized with full weightbearing. Study follow-up visits were scheduled at 3 months (FU1; mean 98 days, SD 10 days) and 1 year (FU2; mean 381 days, SD 23 days).

Longitudinal migration and varus−valgus alignment of the femoral stem were analyzed on anteroposterior (AP) radiographs taken immediately after surgery and at FU1 and FU2 by a single examiner using Wristing^®^ software and the associated technique described in a recent study [7].

Since these measurements can be influenced by rotational positioning of the proximal femur during AP radiographs and DEXA, hip joint positioning aids were routinely used.

According to the systemic measurement error defined by the Wristing^®^ digital software, significant migration or tilt change of the femoral stem was defined as a difference of at least 2 mm or 3°, respectively [15].

AP radiographs of the affected hip taken preoperatively and at FU1 were evaluated to compare caput-collum-diaphyseal (CCD) angle, center of rotation (COR), and offset according to the method described by Lecerf et al. [16].

To evaluate leg length discrepancy (LLD), AP whole pelvis films taken preoperatively were compared with postoperative films (taken prior to hospital discharge). The distance from the tip of the lesser trochanter to the line between the ischial spines (perpendicular) was measured and the difference was calculated. An increased postoperative length value was marked with a plus and a decrease with a minus [17].

AP radiographs were used to evaluate the incidence of periprosthetic radiolucent lines (RL), which were then correlated with Gruen zones at FU2 [18]. RLs were defined as areas of radiolucency at least 1 cm long and 1 mm wide between the prosthesis and the surrounding bone [19].

**Fig. 1:**
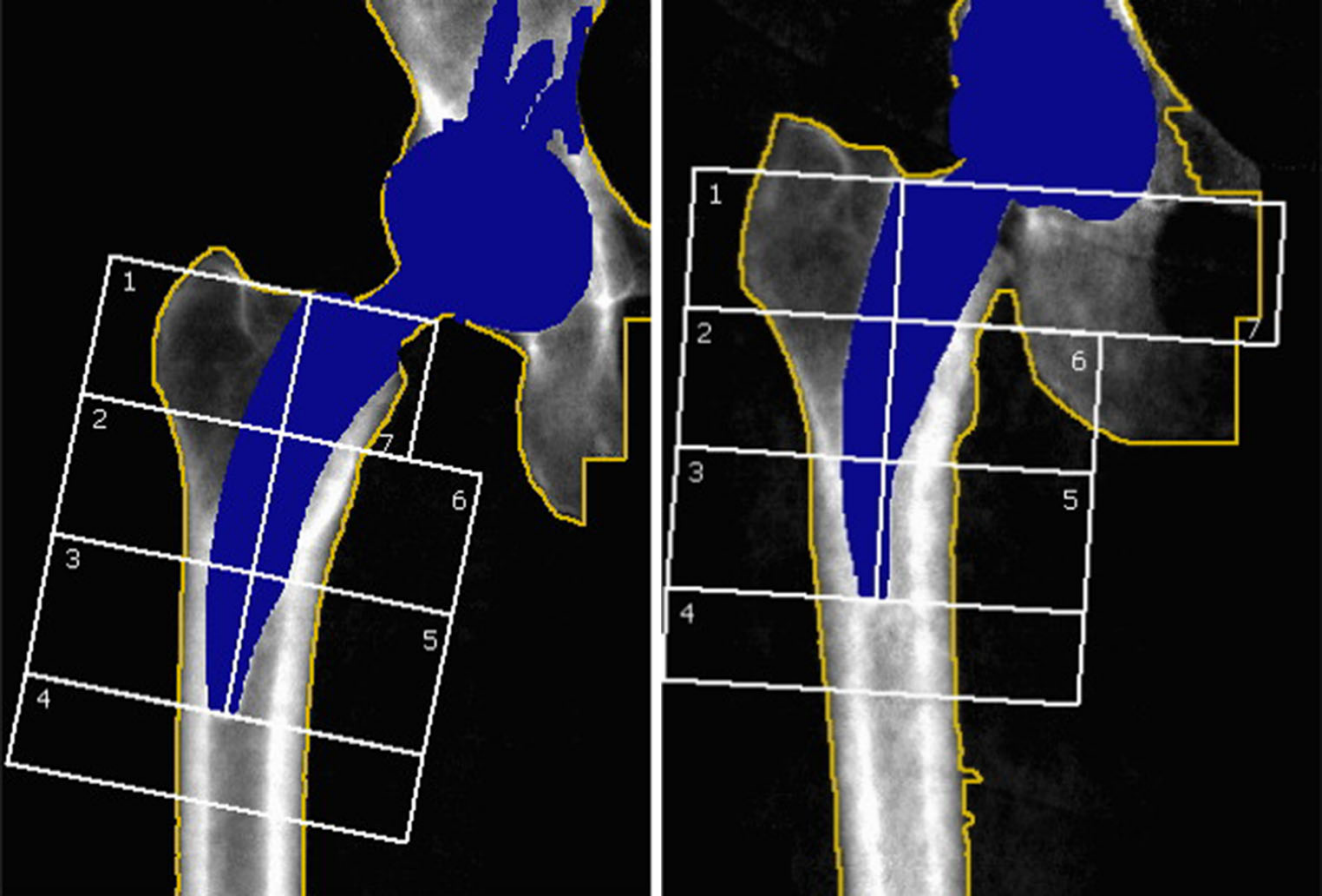
Example of DEXA of the Nanos™ (*right*) and Metha™ (*left*) prosthesis with defined modified Gruen zones

DEXA scans with Gruen zone analysis were performed immediately after THA and at FU1 and FU2 (Lunar DPX- L Fa; Lunar Corp., Wisconsin, USA) (Fig. 1).

Clinical results were evaluated using a visual analog scale (VAS) and the Harris Hip Score (HHS) preoperatively and at FU1 and FU2.

For statistical analysis, unpaired and paired *t*-tests as well as the chi-squared test were used (SPSS 19.0, IBM Company).

## Results

The Metha™ and Nanos™ groups did not significantly differ according to demographic and perioperative data (Table 1).

Significant longitudinal migration was evident in both groups after 3 months, with no significant differences evident between 3 and 12 months postoperatively (paired *t*-test) (Table  2).

**Table 2:** Longitudional migration

	Mean	SD	min–max	*paired t*-test
Metha™ group FU 1	1.87 mm	2.25 mm	0–7 mm	*p* < 0.01
Nanos™ group FU 1	1.96 mm	2.65 mm	0–10 mm	*p* < 0.01
Metha™ group FU 2	1.96 mm	2.37 mm	0–7 mm	*p* = 0.16
Nanos™ group FU 2	2.04 mm	2.65 mm	0–10 mm	*p* = 0.18

SD = standard deviation, min–max = minimum–maximum

CCD measurements showed statistically significantly differences measured pre- and postoperatively (Metha™ = 131° vs 127°; Nanos™ = 130° vs 136°; paired *t*-test, *p* = 0.001). Measurements of COR and off-set did not significantly differ, and were thus regarded as clinically irrelevant.

In addition, both groups exhibited minimal and clinically irrelevant LLDs between the operated and contralateral hips postoperatively. For the Nanos™ group, LLD in the operated contralateral hip averaged 1 mm (min −10 mm, max +6 mm), and for the Metha™ group, the mean LLD measured 0.8 mm (min −2 mm, max +5 mm).

One case in the Nanos™ group resulted in a LLD of 1.0 cm. This is explained by a stem migration of 10 mm at FU1. No further migration occurred between FU1 and FU2. No other radiological or clinical signs of aseptic loosening were present.

Tilt did not significantly change for either Nanos™ or Metha™ stems over follow-up (paired *t*-test, *p* > 0.05).

Areas of radiolucency were detected in 11 cases in the Metha™ group and 8 cases in the Nanos™ group. None of these exceeded a width of 2 mm or length of 1 cm. Therefore, these findings were not considered signs of aseptic loosening [19] and statistical analysis evaluating differences between the groups was not performed.

After 12 months, the DEXA scans showed a very small but significant difference of bone mineral density (BMD) in Gruen zones 1 (approximately −9 %) and 6 (approximately +8 %) for the Metha™ prosthesis (Table 3). For the Nanos™ prosthesis, a significant decrease of BMD was detected in Gruen zone 1 (approximately −14 %; Table 4).

**Table 3:** Metha™ group: results of Dexa

Gruen zone	Postoperative g/cm^2^ (SD)	FU 1 g/cm^2^ (SD)	FU 2 g/cm^2^ (SD)
1	0.86 (0.23)	0.79 (0.26)^*p*=0.001^	0.79 (0.27)^*p*=0.004^
2	1.36 (0.26)	1.42 (0.3)#	1.31 (0.34)#
3	2.22 (0.33)	2.24 (0.33)#	2.13 (0.36)#
4	2.08 (0.35)	2.07 (0.32)#	2.03 (0.36)#
5	1.92 (0.48)	1.94 (0.31)#	1.92 (0.3)#
6	1.5 (0.33)	1.49 (0.31)#	1.62 (0.35)^*p*=0.012^
7	1.28 (0.3)	1.13 (0.28)^*p*=0.004^	1.14 (0.28)#

SD = standard deviation

# Not significant (paired *t*-test, *p* > 0.05)

**Table 4:** Nanos™ group: results of Dexa

Gruen zone	Postoperative g/cm^2^ (SD)	FU 1 g/cm^2^ (SD)	FU 2 g/cm^2^ (SD)
1	0.91 (0.18)	0.83 (0.17)^*p*=0.001^	0.80 (0.17)^*p*=0.005^
2	1.53 (0.32)	1.52 (0.28)#	1.48 (0.23)#
3	2.26 (0.28)	2.23 (0.25)#	2.27 (0.26)#
4	2.14 (0.27)	2.15 (0.36#	2.14 (0.4)#
5	2.17 (0.24)	2.14 (0.38)#	2.15 (0.3)#
6	1.59 (0.3)	1.59 (0.42)#	1.57 (0.37)#
7	1.44 (0.2)	1.31 (0.3)^*p*=0.02^	1.37 (0.3)#

SD = standard deviation

# Not significant (paired *t*-test, *p* > 0.05)

Significant and adequate improvements on VAS and HHS were observed for both implants at 12 months post-surgery. HHS and VAS were 96.5 and 0.7 for the Nanos™ group and 96.2 and 0.8 for the Metha™ group, respectively.

In summary, there was no evidence for aseptic loosening during follow-up.

No intra- or postoperative complications were observed in this study.

## Discussion

The implantation of short-stemmed prostheses has notably increased over the past few years [1–4, 20–25]. In Germany, approximately 15–20 % of primary THAs are now performed using short-stemmed femoral implants.

Possible advantages of short-stemmed femoral prostheses are the reduction of bone loss compared to conventional implants [9, 10, 26–28], their suitability for less invasive surgery, the potential to avoid stress shielding, and theoretically to enable easier revision surgery [1].

Another major point of interest is strain distribution, as this is a precondition for understanding bone remodeling and its impact on the bone quality of the proximal femur. In order to investigate the strain distribution of short-stemmed implants, several studies have been performed, generally based on DEXA scan evaluations. DEXA scans are widely used to evaluate stress shielding and thus indirectly, the force transmission of the prosthetic stem on femoral bone [2, 5, 6, 9, 11–13].

This method is considered an effective way to evaluate BMD over postoperative follow-up, allowing conclusions regarding load transfer induced by the femoral implant [29]. In addition, the reliability of differentiated analysis of BMD according to seven modified Gruen zones after implantation of a femoral implant has been verified [30].

In a prospective randomized trial, Hube et al. (2004) [31] used DEXA scans to compare the osseointegration of the Mayo™ Stem (Zimmer, Warsaw, USA) to that of the ABG™ Prosthesis (Stryker GmbH & Co.KG, Duisburg, Germany) in 93 patients. Approximately 12 months after implantation of the Mayo™ Stem, BMD in the calcar region was increased.

Logroscino et al. (2011) [13] used DEXA scans to evaluate osseointegration of Proxima™ (De-Puy-J&J) and Nanos™ (Smith & Nephew) prostheses. Metaphyseal bone stock was preserved by both implants. Significantly higher BMD values were observed within the metaphysis of the femur with the Nanos™ prosthesis.

In a previous study with a different study group, we investigated bone remodeling and osseointegration of the Nanos™ short-stemmed prosthesis in 25 patients. There were significant decreases of BMD in zones 1, 2, and 7 of 15, 5, and 12 %, respectively, and a significant increase of BMD in Gruen zone 6 of 12 %, which was interpreted as a result of a distally located load transfer and moderate proximally located stress shielding [7].

Lerch et al. (2012) used DEXA scans to validate their finite element (FE) model of strain distribution for the Metha™ stem. To develop the FE model, the law of bone adaptation was used to calculate changes of apparent bone density (ABD) under simulation of physiological loading. They found no difference in ABD or BMD in the distal femur while applying their FE and analyzing the DEXA scans. This finding was interpreted as an absence of stress shielding, which is characteristically found in conventional stems [9].

However, a moderate decrease of BMD was found in the proximal portion of the femur, which was attributed to stress shielding. The considerable remodeling in Gruen zone 6, which contrasts with findings of other study groups [2, 3], was explained by design differences of short-stemmed implants and varying primary rotational stability [6].

In a study examining osseointegration of the Nanos™ prosthesis, Götze et al. (2010) [5] identified bone loss of approximately 7 % in the calcar region and 6 % at the greater trochanter. In contrast to the above-mentioned studies, BMD was significantly increased in Gruen zones 2 and 3, by approximately 10 %. Significant lateral load transfer was present. Thus, the authors concluded that proximal force transmission is not achieved with the Nanos™ prosthesis.

Unfortunately, Götze et al. (2010) did not report on postoperative stem position. According to a previous study, one must consider that valgus positioning of the stem leads to more lateral load transfer and pattern changes of the DEXA. Thus, DEXA results can be affected by different stem positions. The average stem position in the study by Götze et al. (2010) was probably in valgus, which might explain the distal load transfer. To our mind, DEXA results in combination with the Nanos™ stem should particularly be discussed with consideration of the stem position, because the concept of this implant involves an off-set modulation by different implant angulation [5, 7].

In general, studies report decreased BMD of the proximal femur within Gruen zones 1, 2, and 7, and less than what one would expect in conventional THA [9, 10]. This is considered evidence for a moderate distal load transfer.

We found a small to moderate decrease of BMD in Gruen zone 1 for the Metha™ (minus ~9 %) as well as for the Nanos™ stem (minus ~14 %) which supports the conclusion that proximal load transfer occurs for both implants. The Metha™ prosthesis showed an additional increase of BMD in zone 6 (plus ~9 %) that indicates a relevant distal strain distribution. This specific result agrees with the findings of Lerch et al. (2012) [6] who identified a significant BMD loss in Gruen zones 1 and 7 (~10 %), and a BMD increase in Gruen zone 6 for the Metha™ stem (~10 %) after 2 years.

In summary, our study results confirm the conclusions of other investigators who postulated a significant and clinically relevant proximal load transfer for both the Metha™ and Nanos™ stems [3, 6].

These findings suggest only moderate bone loss in the calcar region after implantation of the Metha™ or Nanos™ stems approximately one year postoperatively. For the Mayo™ short-stemmed prosthesis, for instance, a bone loss between 15 % and 18 % has been previously described (4). For conventional THA, proximal BMD loss has been quoted as high as 30 % [10].

The migration of approximately 2 mm after 96 days is not interpreted as a sign of instability of the implants as there was no further migration at the latest follow-up and because of the absence of other signs of aseptic loosening. Furthermore, migration of an implant should not be assumed before a determined difference of 2 mm [32].

We cannot confirm the conclusions in the prospective DEXA study by Goetze et al. (2010) who reported a significant distal load transfer for the Nanos™ implant [5, 7].

In our previous study, we found significant and constant decreases of BMD in zones 1, 2, and 7, of 15, 5, and 12 %, respectively, after 12 months, and a significant increase of BMD in Gruen zone 6 of 12 % [7]. In the current study, there was a significant decrease of BMD of ~10 % in Gruen zone 1 only. There is no plausible explanation for these differences, as the study groups are in the same age group (59.9 [7] vs. 59.8 years), have similar stem position (CCD = 133° [7] vs. 136°), and underwent follow-up DEXA scans at the same time postoperatively (368 [7] vs 381 days). The current study also identified a significant change in BMD in Gruen zone 7 at FU1 (98 days); however, this was not present at FU2.

Lerch et al. described the finding of an increased BMD in Gruen zone 6 for the Metha™ stem as a well-known phenomenon explained by the ‘vast proximal cross section’ of this implant and others like the Mayo™ prosthesis. This circumstance would lead to stress shielding of the proximal portion of the calcar and the greater trochanter, resulting in bone mass decrease. We are convinced that rather than stress shielding, a substantial distally located load transfer is responsible for the moderate loss of BMD in the femoral metaphysis. This conclusion is implied by the interpretation of the law of bone adaptation also used by Lerch et al. [6] for their calculations. In addition, the Mayo™ conservative hip does not feature a ‘vast proximal cross section’. The double-wedge shape of the Mayo™ prosthesis shows a large proximal sagittal diameter compared with other short-stemmed implants such as the Metha™ [33].

Kress et al. [11] suggested that quantitative computed tomography (QCT)-assisted osteodensitometry might be helpful for three-dimensional analysis of the particular remodeling of cortical and cancellous bone around femoral stems. In their study of stress shielding of the C.F.P™ stem (Waldemar Link, Hamburg, Germany), the authors focused on differentiated analyses of BMD changes within cortical and cancellous bone. Because of the different elastic modules of cortical and cancellous bone, they concluded that new prosthetic designs should be validated by in vivo QCT data investigating strain distribution. On the other hand, they conceded that the clinical relevance of such measurements remains to be proven.

The accuracy of DEXA, and its relevance for the assessment of load transfer around femoral implants, has been reported by many others. Lerch et al. [6] pointed out that based on their study results DEXA is an excellent method to analyze bone remodeling after the implantation of short-stemmed prostheses. Cohen et al. [34] concluded that DEXA is a precise method for measurement of small changes in BMD around femoral implants. They indicated that femoral rotation is one of the main causes of failure, and therefore, correct positioning of patients is essential to obtain reliable results. In addition, many other authors have underscored the reliability of DEXA to analyze periprosthetic mineralization processes as a consequence of bone remodeling [9–11, 26–28, 35] or other influences [36]. According to our study protocol, which included the use of positioning aids for DEXA scans, we conclude that the preconditions for precise measurement were present.

One might assume that different stem positions could affect DEXA results. Unfortunately exact stem position has not been reported by other investigators [2, 3, 5, 6, 11, 13]. The law of bone adaptation [37] implies that a particular strain situation induced by different stem positions with variations of off-set and CCD would have consequences regarding the reaction of bone. For example, the Nanos™ short-stemmed prosthesis allows reconstruction of off-set and CCD by different implant positioning, so that bone remodeling should be regarded not only as a prosthesis-specific pattern, but also according to implant position. Therefore, we firmly believe that for short-stemmed implants particularly, the comparability of DEXA studies is limited if stem position is not reported.

The current study has several limitations. One might speculate that follow-up was sufficient to detect changes of BMD. On the other hand, previous studies of conventional stems have concluded that maximum bone remodeling takes place 6 months after surgery and reaches a plateau after ~1 year. Further changes are due to long-term biomechanical adaptation and occur for another 1–2 years. Such changes are minor and show no substantial variation [10, 26]. The size of the study groups is comparable with others [2, 5, 6, 9, 11]. In addition, DEXA measurements are regarded as extremely reliable and unaffected by subjective errors [29].

The radiological measurement of stem migration and angulation was not performed using an established method like EBRA [38]. However, the method used in this study has been validated and successfully performed in other similar investigations [15, 39, 40].

In summary, we conclude that the Nanos™ and Metha™ prostheses show no substantial or clinically relevant differences regarding the reduction or loss of bone in the proximal aspect of the femur. Both stems show excellent clinical results and reliable osseointegration over a short follow-up period.

The moderate BMD changes of the femoral metaphysis are interpreted as a result of the presence of physiological strain distribution [37, 41]. Thus, the concept of a short-stemmed femoral implant with proximal strain distribution is confirmed for both implants.

However, neither of the prostheses was able to completely prevent a certain amount of stress shielding in the calcar and major trochanter regions, which is interpreted as moderate underloading and distal load transfer, respectively. Furthermore, one must consider that evidence is still pending regarding the clinical value of the preservation of proximal bone mass in terms of long-term survival or improved options for revision surgery for these kinds of implants.
